# The 3^rd ^Computational Structural Bioinformatics Workshop

**DOI:** 10.1186/1472-6807-10-S1-I1

**Published:** 2010-05-17

**Authors:** Jing He, Di Wu

**Affiliations:** 1 Department of Computer Science, Old Dominion University, Norfolk, VA 23529, USA; 2 Department of Mathematics and Computer Science, Bioinformatics and Information Sciences Center, Western Kentucky University, Bowling Green, KY 42103, USA

## Summary

As many other domains in biology, molecular structures have proposed challenging but interesting computational problems. The unique challenge of the 3-dimensional molecular structures comes from the combination of the fundamental concepts of physics, chemistry, biology and geometry, and it is often computationally intensive to search for the correct structure. The Computational Structural Bioinformatics Workshop (CSBW) is a workshop that focuses on the fundamental computational work that is related to 3-dimensional molecular structures. This workshop aims to bring together researchers with expertise in bioinformatics, computational biology, structural biology, data mining, optimization and high performance computing to discuss recent results, new techniques, and open research problems in computational structural bioinformatics.

The first workshop was held on November 4, 2007 in San Jose California, in conjunction with the International Conference on Bioinformatics and Biomedicine (BIBM). The papers in this issue were selected from the third workshop that was held on November 1, 2009 at Washington D.C. We received thirty-three paper submissions in 2009. Eight were selected for an extended version for the publication on *BMC Structural Biology*. Although the number of the submissions is limited, the papers often come from the research groups that have the established records in structural bioinformatics. The next CSBW is going to be at Norfolk Virginia. We will continue our effort and hold the next workshop even better.

## Competing interests

The authors declare that they have no competing interests.

